# Long term repeated fire disturbance alters soil bacterial diversity but not the abundance in an Australian wet sclerophyll forest

**DOI:** 10.1038/srep19639

**Published:** 2016-01-20

**Authors:** Ju-pei Shen, C. R. Chen, Tom Lewis

**Affiliations:** 1 Environmental Futures Research Institute and Griffith School of Environment, Griffith University, Nathan QLD 4111, Australia; 2 Research Centre for Eco-Environmental Sciences, Chinese Academy of Sciences, Beijing, China; 3 Horticulture and Forestry Science, Department of Agriculture, Fisheries and Forestry, University of the Sunshine Coast, Sippy Downs Drive, Sippy Downs, QLD 4556, Australia

## Abstract

Effects of fire on biogeochemical cycling in terrestrial ecosystem are widely acknowledged, while few studies have focused on the bacterial community under the disturbance of long-term frequent prescribed fire. In this study, three treatments (burning every two years (B2), burning every four years (B4) and no burning (B0)) were applied for 38 years in an Australian wet sclerophyll forest. Results showed that bacterial alpha diversity (i.e. bacterial OTU) in the top soil (0–10 cm) was significantly higher in the B2 treatment compared with the B0 and B4 treatments. Non-metric multidimensional analysis (NMDS) of bacterial community showed clear separation of the soil bacterial community structure among different fire frequency regimes and between the depths. Different frequency fire did not have a substantial effect on bacterial composition at phylum level or bacterial 16S rRNA gene abundance. Soil pH and C:N ratio were the major drivers for bacterial community structure in the most frequent fire treatment (B2), while other factors (EC, DOC, DON, MBC, NH_4_^+^, TC and TN) were significant in the less frequent burning and no burning treatments (B4 and B0). This study suggested that burning had a dramatic impact on bacterial diversity but not abundance with more frequent fire.

Forest ecosystem is among the most heterogeneous and complex environments on the planet. Soil microbes represent a considerable fraction of forest ecosystem, regulating nutrient transformations and energy flow in the soil. Soil microbial diversity is of critical importance in maintaining the sustainability of ecosystem and has suffered greatly due to anthropogenic disturbances, such as fertilization^1^,^2^, fire^3^ and deforestation^4^. Such disturbances may cause shifts in microbial composition resulting in substantial impacts on biogeochemical processes and ecosystem function^5^. Understanding biotic response to these disturbances is thus critical in predicting the consequences of long-term human-induced changes in forest ecosystem^6^.

Fire is one of the main concerns of global climate change. Many studies have documented the fire effects on forest ecosystem including enhancing plant productivity^7^, shifting in plant community structure^8^, accelerating carbon (C) cycling process^9^ and reduction of fungal and bacterial biomass^10^. However, the impacts of fire on forest ecosystem varied greatly with the differences in its intensity, frequency, forest type, the slope, fuel load and type and environmental conditions (e.g. moisture and temperature) as well as the characteristics of different microbial groups (e.g. sensitivity to fire)^11^. For example, a meta-analysis based on 42 wildfire studies demonstrated that wildfires lead to a more significant reduction in microbial biomass compared to managed burning^12^. Prescribed fire is one of the common practices reducing the risk of wildfire occurrence, which in contrast causes extensive damage to forest vegetation and soils^13^. Extensive studies have demonstrated the effectiveness of prescribed fire in wildfire hazard abatement as well as the mitigation of CO_2_ emissions from forest fires, while little information is available about the impacts of long-term different fire frequencies on soil microbial community in terms of bacterial diversity and abundance^14^,^15^, especially individual bacterial taxonomic change^16^.

Anthropogenic disturbance such as land use conversion, climate change and altered fire regimes, could have major consequences for microbial genetic resources, which in turn influences ecosystem properties like resistance and stability^17^,^18^,^19^. However, the degree to which specific microbial groups is influenced varies with the microbial sensitivity or recovery capacity when subjected to perturbations^16^,^20^. For example, bacterial community composition recovered rapidly and returned to the similar condition as no fire interference after burning for 11 years^3^. Furthermore, microbial community assembly mainly are driven by neutral processes while changed to niche process along the time since fire disturbance^21^. On the other hand, as fire effect is spatially heterogeneous within one fire event or one area of interest, due to its patchy nature (i.e. mosaics), which could largely influence the relative contribution of niche and neutral processes ascribing to the uneven distribution of fuels, soil moisture and micro-geographical condition (e.g. slope)^11^,^15^. Additionally, as disturbance (e.g. prescribed fire) becomes frequent and exerts continuous pressure, severe implications may be apparent and thereby could cause long-term cumulated negative impacts on key biogeochemical processes^22^. Therefore, it is of critical importance to understand the role of recurring fire disturbance for microbial groups and environmental assessment^23^.

It is generally difficult in determining the disturbance effects, which are normally confounded with other factors. Therefore, it is critical to find out the key factors as well as the ecological process driving microbial community composition. Pyrosequencing techniques has shown to be a powerful tool in discovering microbial diversity and community composition via deciphering the distribution of microbial groups and their interaction with abiotic factors^21^,^24^,^25^. We hypothesized that more frequent fire disturbance would have a greater impact on soil bacterial diversity compared to less frequent one due to differences in soil parameters mediated by fire. The objectives of this study were, therefore, to i) determine the effects of long-term prescribed fire on the diversity of bacteria; and ii) identify the main factors contributing to alterations of the bacterial community composition using pyrosequencing technique targeting 16S rRNA region.

## Results

### Soil chemical properties

Soil chemical properties were greatly impacted by frequent fire at the Peachester site (Table 1). At the depth of 0–10 cm, soil total C (TC), total N (TN), moisture, dissolved organic C (DOC), dissolved organic N (DON) and microbial biomass C (MBC) were all significantly lower in the more frequent fire treatment (B2) than in the less frequent (B4) and no-burning (B0) treatments. The highest soil pH value (ca. 5.41) was recorded in the B2 treatment, followed by the B4 treatment (5.11) and B0 treatment (4.86), while electrical conductivity (EC) showed the opposite trend (Table 1). No significant difference among the treatments was detected at the 10–20 cm depth for all the measured soil properties, except soil moisture. Some of the resource-related variables (i.e. TC, TN, DOC, DON, NH_4_^+^, MBC and microbial biomass N (MBN)) at the 10–20 cm depth were significantly lower compared to the 0–10 cm depth for B4 and B0 treatments, but no differences between depths were observed for the B2 treatment except for DOC (Table 1). There was no significant difference in the inorganic phosphorus (Pi), C:N ratio and nitrate (NO_3_^−^) irrespective of depth or treatment though the variance was high for nitrate.

### Soil bacterial diversity and the relationship with soil environmental properties

**Figure 1:**
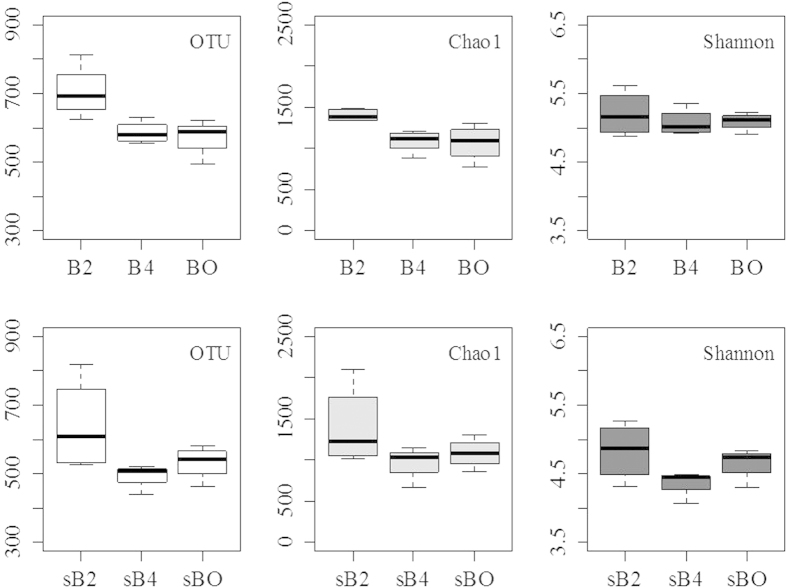
Boxplot of bacterial alpha-diversity indices for the 16S rRNA gene sequences among different treatments at two depths. B0, B2 and B4 represent the 0–10 cm samples from the treatments of no burning, burning every two years and burning every four years, respectively, while sB0s, sB2 and sB4 represent the 10–20 cm samples. Top two panels are bacterial diversity indices derived from soil samples at the depth of 0–10 cm, and bottom two panels are at the depth of 10–20 cm. Fire effects across treatments indicated by asterisks: *P < 0.5.

In total, 295,185 quality sequences were obtained after eliminating one sample with 411 sequences, resulting into 2,753 to 18,210 sequences per sample (Table S1). Bacterial community diversity was calculated based on the relative abundance of OTU. In the top soil the most frequent fire treatment (B2) had significantly higher bacterial alpha-diversity relative to the other treatments, regardless of the parameters (i.e. OTU, Chao1) based on OTU matrix (Fig. 1). No significant difference between treatments was detected for the Shannon indice. There were no significant treatment effects in all alpha-diversity indexes measured for the 10–20 cm depth. A similar trend of highest diversity in the B2 treatment was also observed by rarefaction curves as measured by OTU richness with average values for triplicate treatments irrespective of sample size (Fig. S1 A and B).

**Figure 2:**
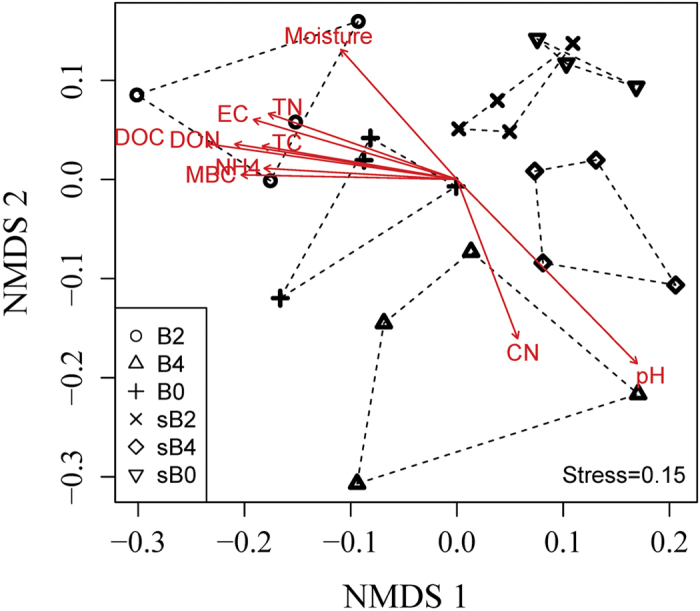
Non-metric multidimensional scaling plot of soil bacterial community structure in three fire treatments and at two depths using OTU-based Bray-Curtis dissimilarities distance with soil chemical properties. B0, B2 and B4 represent the 0–10 cm samples from the treatments of no burning, burning every two years and burning every four years, respectively, while sB0s, sB2 and sB4 represent the 10–20 cm samples. Only soil chemical properties with *P* values less than 0.5 are plotted, and the arrow length is proportional to the strength of correlation. Stress value is also indicated in the figure. For abbreviations for soil chemical properties refer to Table 1. The R^2^ value for stress test is 0.976 (non-metric fit) and 0.875 (linear fit).

Non-metric multidimensional scaling clustered soil samples by depth and treatment (*P* = 0.0099, Monte carlo randomization test) (Fig. 2). The most frequent burning treatment (B2) was clearly separated from the other treatments (B4 and B0) within each depth. The separation of community structure was supported by an analysis of similarity of bacterial communities (ANOSIM) for different treatments at each depth (Table 2). ANOSIM of Bray-Curtis and UniFrac distance dissimilarities for depth and frequent fire effects both showed significant differences (*P* < 0.05), but there was no difference between the burnt (B2 and B4) and unburnt (B0) treatments (*P* > 0.05) (Table 2).

A Mantel test further revealed the relationship between soil bacterial community structure and abiotic factors under the different frequent fire treatments. There were significant correlations (*P* < 0.01) between soil properties (i.e. DOC, DON, pH, EC and NH_4_^+^) and bacterial community composition generated by Bray-Curtis (OTU-based) dissimilarities (Table 3). In order to eliminate bias caused by rare OTUs, we also calculated the correlation between soil properties and the dominate OTUs (66 OTUs), and this analysis revealed the similar trends to those calculated using most OTUs (Table 3).

### Bacterial 16S rRNA gene composition and abundance

**Figure 3:**
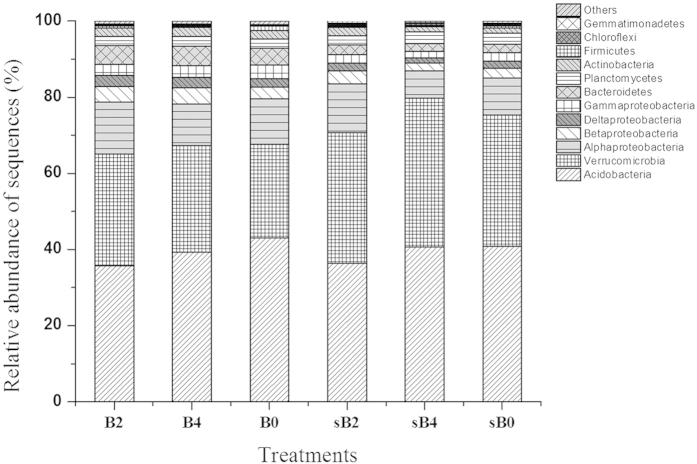
Percent community composition of numerically dominant bacterial phyla across three fire treatments at two depths.

**Figure 4:**
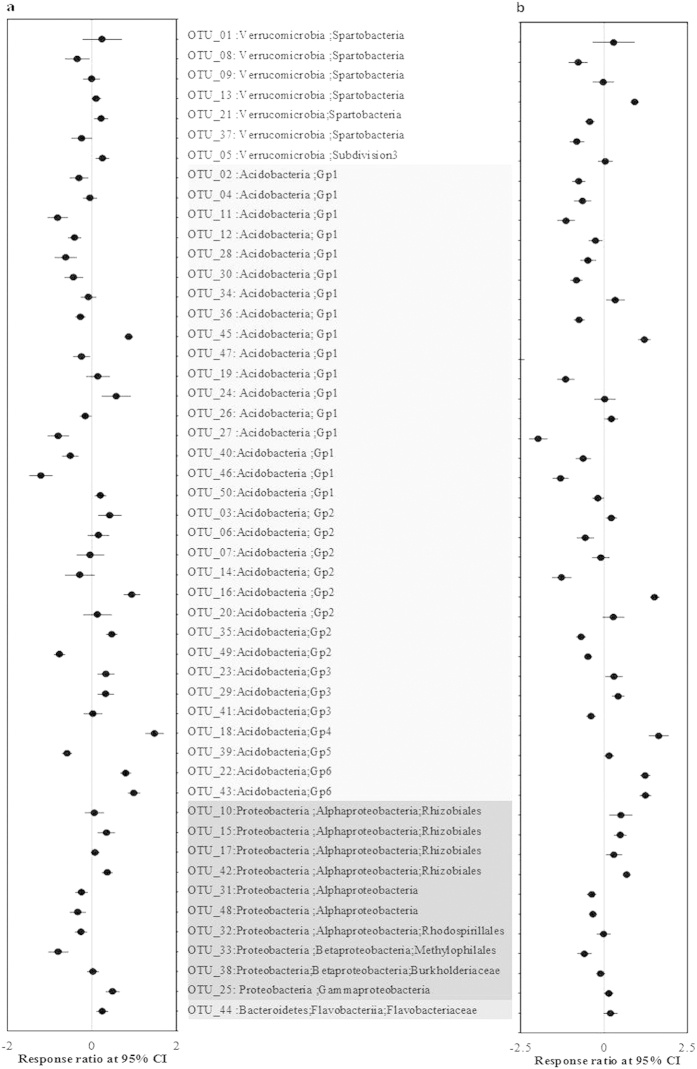
Response ratios of (**a**) burning every 4 years to no burning treatment and (**b**) burning every 2 years to no burning treatment. Significance was determined at a 95% CI (confidence interval).

A taxonomic summary of bacterial composition showed that all soils were typically dominated by *Acidobacteria*, ranging from 36% to 42% of all sequences across the treatments (Fig. 3). More interestingly, phylum *Verrucomicrobia* appeared to be the second most dominant group (24–39%), which was more abundant than the *Proteobacteria* group (12–23%) (t_23,23_ = 4.7, *P* < 0.001) (Fig. 3). The relative abundance of *Verrucomicrobia* was much higher at the 10–20 cm depth compared to the top soil (0–10 cm), while *Alphaproteobacteria* and *Bacteroidetes* showed an opposite trend along the depth (Fig. 3). There was no significant difference in the relative abundance of dominant phyla among treatments using Chi-Square tests. However, significant responses were detected for the most abundant OTU at lower levels of taxon between burning and non-burning treatments (Fig. 4). For example, the most detected subgroup Gp1 *Acidobacteria* had a significantly lower abundance except OTU_45 in response to burning (Fig. 4). For *Alphaproteobacteria*, in the order *Rhizobiales*, most OTUs showed higher abundance especial to the most frequent burning (Fig. 4b). Similarly, by comparing to no burning treatment, *Verrucomicrobia* taxa showed a highly variable response in burning every two years (B2) than every four years (B4). Spearman’s correlations were measured between the relative abundance of the main phyla and soil chemical properties to assess the relative contribution of biotic and abiotic factors on the bacterial community composition. *Verrucomicroia* and *Chloroflexi* were negatively correlated with some soil properties (i.e. DOC, DON, MBC and MBN), while *Gammaproteobacteria* and *Bacteroidetes* were positively correlated with most of the soil properties (Table S2).

As detected by real-time PCR targeting 16S rRNA gene, bacterial abundance were recorded from 4.67 × 10^9^ in the B2 treatment to 2.96 × 10^9^ copy numbers per gram dry soil in the sB4 treatment. There was no significant difference in soil bacterial 16S rRNA gene abundance across the treatments or the depths (Fig. S2). This result is consistent with the bacterial 16S rRNA gene quantitative results of soil samples taken from the same site in 2005^26^ (Fig. S2).

## Discussion

Fire has modified majority of earth surface and it has not only affect the aboveground species composition, but also the belowground community^5^. In this study, NMDS analyses based on pyrosequencing data showed that soil bacterial communities in the most frequently burnt treatment were significantly different from those in the less frequently burnt and long unburnt treatments, especially in the topsoil. Furthermore, a significant increase in bacterial alpha diversity (i.e. OTU and Chao1) was detected in topsoils of the most frequent burning treatment (B2), which is congruent with the observations for fungal communities at the same site^27^ and with a study of similar fire regimes in the United States^28^. ANOSIM analysis based on the Bury-curtis and Unweighted UniFrac distances also showed that bacterial community structure was significantly affected by the most frequent fire treatment as well as depth. The increase in bacterial community diversity in response to frequent fire may be partly attributable to the spatial heterogeneity of fire effects caused by the inherent patchiness of low intensity fire due to uneven distribution of fuels, slope position and soil moisture in wet eucalypt forest^11^,^29^. Fire disturbance would increase clumping of fire-intolerant species, such as in frequently burned pine savannas of southeastern North America, resulting in higher β-deviations as well as the stronger link with environmental factors in burning treatments comparing to non-burning^30^. Microbial diversity tended to increase following wildfire disturbance over time^21^. Fire disturbance creates heterogeneity in forest soil properties that may maintain and even improve the bacterial diversity by affecting the competition ability of bacteria for resources^31^,^32^,^33^. This phenomenon is compatible with Connell’s intermediate disturbance hypothesis proposing that maximum levels of diversity are obtained at intermediate frequencies of disturbance as corroborated by previous studies^34^. Indeed, more recent research focusing on diversity-disturbance relationships has found positive relationships between diversity and both disturbance intensity and frequency^35^, which has further corroborated our finding.

The observed shift of bacterial community structure was more directly attributable to the change of soil chemical factors as evidenced by the significant correlation between bacterial community structure and selected soil properties with Mantel test using OTU-based dissimilarity (Table 3). Among the selected variables, soil pH was one of the major factors contributing to the observed bacterial community variations, showing a positive correlation with frequent fire treatments. Fires, whether prescribed or wildfire, can increase soil pH due to the deposition of ash^20^,^36^, and therefore drive a shift in the bacterial community structure^37^. Accumulating literature has already documented this pH-driver phenomena for different microbial groups, including bacteria^38^, fungi^39^, and some specific taxa *Acidobacteria*^40^. However, it is still unclear whether communities were influenced directly by pH or indirectly through influencing other factors altered by pH^37^. Other research on fire regimes suggested that microbial structure is largely related to the availability of substrates, but not soil pH^20^. Furthermore, it has been suggested that the reduction of total C and N stocks contributed to the shifts of the general fungal and ectomycorrhizal communities at the same site^27^,^41^. As such, we proposed that soil pH was the main factor for the shift of bacterial community structure under the most frequent fire disturbance (e.g. B2), while for those treatments with little variation in soil pH (i.e. B4 and B0), resource availability mainly attributed to the distribution pattern of bacteria.

In contrast to bacterial community structure, little variation of bacterial abundance was observed across the fire treatments in this study based on quantitative analysis of 16S rRNA gene using real-time PCR technique. This finding is consistent with earlier studies at the same site for the species richness of *Basidiomycete* fungal communities^27^ and a seasonal comparison of bacterial 16S rRNA gene copies in 2011^42^. When combined with results from previous measurements for bacterial 16S rRNA gene copies from 2005 and 2011, all showed a consistent trend (i.e. no significant difference among fire treatments) using real-time PCR technique^1^,^42^. The real-time PCR technique is a robust method and has been widely used in microbial abundance analysis. However, it cannot be ruled out that variation in the abundance of specific bacterial groups at lower taxonomic levels may occur in response to fire. Furthermore, the variable impacts of frequent fire on bacterial diversity and relative abundance at phylum level were largely driven by the less frequent taxa that responded rapidly to recurring fire disturbance^28^,^43^. Indeed, a comparison of unique OTUs and relative abundance of sequences for each dominant bacterial taxon further supported this finding (Fig. S3). The classification of bacterial groups was recently been proposed as copiotrophic and oligotrophic according to nutrition and growth^44^. This ecological feature of bacterial phyla was largely related to the variation in relative abundance of dominant taxa following exotic disturbance^45^,^46^. *Bacteroidetes* and *Proteobacteria*, for instance, are typically copiotrophic and respond rapidly to the availability of resources, such as soluble carbon^44^,^47^. This is supported by the positive relationship between relative abundance for these two groups and soil resource variables (i.e. DOC, DON, NH_4_^+^, MBC and MBN) in our study. In contrast, an unexpected higher abundance of *Verrucomibia* was observed, with negative correlations with soil variables DOC, DON, MBC and MBN, suggesting their slow-growing life strategy is favoured by low resource habitats^48^.

This study clearly demonstrated for the first time that significant alteration of bacterial community diversity and structure by the long-term frequent fire disturbance. Biennial fire greatly changed bacterial community structure, while minimal effects on bacterial composition and abundance. Moreover, this study has also shown the dominance of taxa *Verrucomicrobia*, implying that this group may play an important role in biogeochemical processes, such as C cycling^49^. Further work on its contribution to ecosystem function is required to better understand the response of microbial communities to frequent and severe disturbances. Moreover, future work also needs to look into the time series of microbial community pattern in order to predict the response of microbial groups as well as assess the recover capacity for recurring disturbances.

## Methods

### Sampling site

Our experimental site is located in Peachester State Forest, southeast Queensland, Australia (26°50′S, 152° 53′E), which has been described in detail previously^42^,^50^. In brief, three prescribed burning treatments, i.e. burning every two years (B2), burning every four years (B4) and no burning (B0), were established in 1972, and low intensity fires (<2,500 kWm^−1^) were carried out in the winter. The Peachester site has an average annual rainfall of 1,711 mm and average minimum and maximum temperatures of 14 °C to 24 °C, respectively. It is a typical wet sclerophyll forest and the vegetation is dominated by *Eucalyptus pilularis* Smith with lesser proportions of other canopy species including *Corymbia intermedia*, *Eucalyptus microcorys*, *Eucalyptus resinifera*, *Syncarpia glomulifera* and *Lophostemon confertus*. Our previous research in this experiment site has demonstrated that long-term repeated burning had significant impact on vegetation community structure and composition^51^. Soil samples were taken at depths of 0–10 cm and 10–20 cm in July 2010, before the next scheduled burning (August 2010) for the B2 and B4 treatments. Fifteen soil cores were randomly taken from each plot and bulked as a composite sample, and in total 24 composite samples (across two depths) were collected from four plots (replicates) in each treatment. Soil samples were passed through a 2.0- mm sieve after removing root debris and stones, and kept at 4 °C for chemical and biological analyses. Subsamples were kept at −80 °C for further analysis.

### Soil chemical properties analysis

Soil moisture was determined gravimetrically via drying the soil at 105 °C for 24 h, and methods for soil chemical properties have been described in previous research^52^. Soil pH and electrical conductance (EC) were measured in the ratio of 1:5 (soil-to-water) with a pH/EC meter. Microbial biomass C (MBC) and N (MBN) were measured by the chloroform fumigation extraction method. Soil samples were extracted using 2 M KCl solution for analysis of ammonium (NH_4_^+^-N) and nitrate (NO_3_^−^-N) with Westco Smart Chem Discrete Wet Chemistry Analyzer (Westco Scientific Instruments, USA). Soil total C (TC) and N (TN) content were measured with a Eurovector Elemental Analyser (Isoprime-EuroEA 3000, Milan, Italy). Dissolved organic carbon (DOC) and total dissolved organic nitrogen (DON) were extracted by deionized water (1:5, soil:water) via incubating at 70 °C for 16 hours, then measured with a TOC-VCPH/CPN analyser fitted with a TN unit (Shimadzu Scientific Instruments, Columbia, USA).

### Soil DNA isolation and pyrosequencing

The total genomic DNA was extracted from 0.3 g of frozen soil using MoBio Powersoil DNA isolation kit following the manufacture’s instruction. 10-fold dilution of DNA template was used for downstream PCR analysis to reduce PCR inhibitors such as humic acid. PCR products for pyrosequencing were amplified with the bar-coded primers F515 (5′-GTGCCAGCMGCCGCGGTAA-3′) and R806 (5′-GGACTACVSGGGTATCTAAT-3′), targeting the V4 region of the bacterial and archaeal 16S rRNA genes^53^,^54^. The forward primer consisted of the 454 adapter A, a 10-based MID barcodes, 4-bp linker sequence (TCAG), along with the primer F515. The reverse primer consisted of the 454 adapter B, 4-bp linker sequence (TCAG) along with the primer R806. PCR amplification were performed in triplicate in 30 μl reaction containing 1 × PCR buffer, 3.0 mM MgCl_2_, 400 μM each dNTP, 2.5 U Ex Taq^TM^ DNA polymerase (TaKaRa, Shiga, Japan), 0.5 μM of each bar-coded primer. We used the following PCR thermal profile: 94 °C for 5 min, 34 cycles consisting of 95 °C for 30 s, 53 °C for 45 s, 72 °C for 60 s, and final extension at 72 °C for 60 s. Pooled PCR products for each soil sample were purified using a Wizard SV Gel and PCR Clean-Up System (Promega, Madison, WI). The purity and concentration were checked using a NanoDrop 1000 spectrophotometer (Thermo Scientific, Wilmington, DE) and Qubit fluorometer with Quant-iT dsDNA HS Assay Kits (Invitrogen, Eugene, OR), respectively. Equimolar amounts of PCR products with different barcodes were combined into one sample and sent to Macrogen (Seoul, Korea) for pyrosequencing using Roche/454 GS FLX Titanium platform.

In total, 43, 8678 raw sequences were obtained from 24 samples. Mothur 1.30.1 software^55^ was employed to process 454 raw data. Sequences were trimmed and aligned for quality check with Mothur pipeline after deionising with the command of Shhh.flows, following the main steps of previous research^56^. Sequences with eight or more homopolymers and two or more ambiguous bases were removed. Filtered sequences were aligned against the SILVA reference database^57^ for chimera check using chimera.uchime function^58^. There were 7,654 singleton sequences removed before final diversity analysis^59^. The bacterial sequences were eventually binned into operational taxonomic units (OTU) to generate a rarefaction curve and to calculate species richness estimators at the sequence identity level of 97% using the average neighbor method. OTU as a measure of a single sample alpha diversity indicates the richness that the numbers of OTUs are observed. Rarefaction curve is one of the commonly used species richness estimators to determine whether sampling depth is sufficient to accurately characterize the bacterial community^60^. The Chao 1 index as another commonly used species richness estimator will also be used in our study based on the number of rare OTUs in a sample^61^. The Shannon index accounts for both abundance and evenness of the species present, which is not sensitive to sample size. The taxonomic assignment of representative sequences was achieved using the Ribosomal Database Project (RDP) Classifier^62^ with a minimum confidence of 80%.

Alpha diversity was calculated based on the relative abundance of OTU. In order to minimize the variation caused by the sampling sequences, each sample was rarefied to the minimum sequence number (2,753 sequences) using subsample argument in Mothur.

Quantitative PCR for 16S rRNA gene was carried out using primer pair 338F/518R, and PCR thermal profile and standard curve preparation followed the procedure as described in detail by Liu *et al.*^42^. Amplification efficiency for 16S rRNA gene was 95–98% with R^2^ values greater than 0.99 for the standard curve.

### Pyrosequencing data analysis

In total, 295,596 quality sequences were obtained from 24 samples with the fragment length ranging from 235 to 313 bp. However, one of the samples from the burning every four years (B4) with the minimum number of sequences (i.e. 411) was eliminated for subsequent analysis of alpha and beta diversity, eventually, resulting into 2,753–18,210 sequences per sample after data trimming and quality control (Table S1). There were 35.2%, 22.5%, 11.20%, 10.49%, 6.20% and 6.19% of OTUs affiliated with *Proteobacteria, Acidobacteria, Planctomycetes, Verrucomicrobia, Actinobacteria* and *Bacteroidetes* (Fig. S3 bottom panel), respectively. These six phyla contributed to 91.8% of total OTUs. Approximately 0.7% OTUs could not be assigned to any phylum. Of all the assigned OTUs at 97% similarity, the most abundant OTU belonged to *Verrucomicrobia*, which accounted for 20.4% of all the sequences.

### Statistical analysis

Before performing ordination and correlation analysis based on the OTU matrix, those OTUs with less than five detected sites were eliminated in order to achieve a robust result (eventually 736 unique OTU were selected). Relationships between bacterial community distribution data and soil chemical parameters were evaluated with a Mantel test using OTU-based Bray-Curtis distance matrices with 999 permutations in R software Vegan v2.0–8 package. Euclidean distance was used to generate a soil chemical parameters distance matrix. Ordination and then the relationship of these data with soil chemical parameters was estimated using *evfit* function of Vegan v2.0–8 library with 999 permutations. The accuracy of the two-dimensional representation is indicated by the “stress” value. Distances between symbols represent relative dissimilarity between communities. We also did a Monte Carlo test to check the statistical significance of the generated two dimensions (k = 2). To determine if any group of samples contained significantly different bacterial communities, the analysis of similarities (ANOSIM) function was used with Bray-Curtis (OTU-based) dissimilarity matrices and UniFrac (Phylogenetically-based) distance using the statistical software R, Vegan package^63^. All the tests were performed using R v2.15.3 statistical program (http://www.r-project.org).

One-way ANOVA was used for comparison of bacterial diversity indices using SPSS 16.0. All values including soil chemical properties were log or square-root transformed to meet test assumptions. Spearman’s rank correlation was calculated to compare the relationship within soil chemical properties and with the composition of taxa associated with fire treatments using SPSS Bivariate correlations. To test heterogeneity in frequency of taxon among different burning treatments, chi-square tests were performed with the relative abundance of dominant phyla using SPSS 16.0. Response ratio was applied to analyse the effects of different frequent fire on the main dominant OTU (the 50 most dominant OTUs) compared to non-burning site using the calculation methods as suggested by Luo *et al.*^64^ and Liang *et al.*^65^. Significance was determined at a 95% CI (confidence interval).

## Additional Information

**Accession codes:** The sequence data have been submitted to the GenBank database under SRA accession number SRP032649. Sample site and barcode information are both associated with NCBI SRA submission.

**How to cite this article**: Shen, J.- *et al.* Long term repeated fire disturbance alters soil bacterial diversity but not the abundance in an Australian wet sclerophyll forest. *Sci. Rep.* **6**, 19639; doi: 10.1038/srep19639 (2016).

## Supplementary Material

Supplementary Information

## Figures and Tables

**Table 1:** Soil chemical properties across the burning treatments.

Treatment	TC	TN	Pi	C:N	Moisture	DOC	DON	pH	EC	NO_3_^−^	NH_4_^+^	MBC	MBN
B2	3.15 ± 0.37(b)*	0.12 ± 0.02(b)	24.6 ± 4.2(a)	26.0 ± 2.5(a)	9.99 ± 1.73(b)	389 ± 77(b)	21.7 ± 5.9(b)	5.41 ± 0.14(a)	25.2 ± 2.1(b)	5.7 ± 3.5(a)	9.2 ± 2.09(ab)	391 ± 28(b)	63.6 ± 14.1(abc)
B4	4.93 ± 0.70(a)	0.23 ± 0.02(a)	23.0 ± 5.0(a)	21.5 ± 1.6(a)	18.8 ± 1.2(a)	672 ± 86(a)	37.1 ± 5.3(a)	5.11 ± 0.27(ab)	34.2 ± 15.4(ab)	14.5 ± 13.6(a)	11.0 ± 1.8(a)	529 ± 10(a)	84.3 ± 18.4(a)
B0	4.75 ± 1.45(a)	0.22 ± 0.08(a)	26.0 ± 6.5(a)	21.7 ± 2.3(a)	17.4 ± 5.5(a)	676 ± 69(a)	37.3 ± 9.5(a)	4.86 ± 0.06(b)	41.4 ± 7.2((a)	20.2 ± 12.0(a)	12.0 ± 2.2(a)	513 ± 9(a)	75.2 ± 9.9(ab)
sB2	2.31 ± 0.19(b)	0.09 ± 0.01(b)	22.3 ± 5.5(a)	25.2 ± 3.5(a)	9.05 ± 1.36(b)	240 ± 61(c)	14.1 ± 6.3(b)	5.43 ± 0.04(a)	22.7 ± 3.8(b)	7.6 ± 4.9(a)	7.3 ± 1.7(b)	320 ± 65(b)	55.4 ± 15.0(bc)
sB4	3.38 ± 0.51(b)	0.15 ± 0.02(b)	16.8 ± 7.3(a)	22.8 ± 1.9(a)	15.6 ± 0.9(a)	280 ± 20(bc)	13.7 ± 2.3(b)	5.24 ± 0.24((a)	22.4 ± 3.5(b)	8.5 ± 7.5(a)	6.8 ± 0.7(b)	293 ± 61(b)	51.4 ± 14.9(c)
sB0	3.26 ± 0.85(b)	0.15 ± 0.04(b)	23.7 ± 2.9(a)	22.3 ± 2.1(a)	14.2 ± 4.5(ab)	289 ± 61(bc)	13.7 ± 2.4(b)	5.11 ± 0.08(ab)	24.9 ± 5.1(b)	12.6 ± 5.2(a)	7.2 ± 1.1(b)	292 ± 39(b)	44.0 ± 5.9(bc)

^*^The different letter at the same column indicated significant difference at *P* < 0.05.

The unit % for TC, TN and moisture; mg kg^−1^ for Pi, DOC, DON, NO_3_^−^, NH_4_^+^, MBC and MBN; and μs cm^−1^ for EC.

Abbreviation: TC, total carbon; TN, total nitrogen; Pi, NaHCO_3_-extractable inorganic phosphorus; C:N, the ratio of TC to TN; DOC, hot water extracted dissolved organic carbon; DON, hot water extracted dissolved organic nitrogen; EC, electrical conductance; MBC, Microbial biomass carbon; MBN, microbial biomass nitrogen.

B0, B2 and B4 represent the 0–10 cm samples from the treatments of no burning, burning every two years and burning every four years respectively while sB0s, sB2 and sB4 the 10–20 cm samples from the same treatments.

**Table 2:** Results of ANOSIM tests performed on OTU-based Bray-Curtis distance and phylogenetic -based UniFrac distance.

Metric	Variable of interest	Partial predictor	r	*P*
Bray-Curtis	Treatment	Depth	0.514	**0.002**
	Depth	—	0.4841	**0.001**
	Fire*	Depth	0.0700	0.114
	Freq fire	Depth	0.3242	**0.001**
UniFrac-UW	Treatment	Depth	0.3906	**0.002**
	Depth	—	0.3227	**0.001**
	Fire	Depth	0.0854	0.148
	Freq fire	Depth	0.4613	**0.001**

Analyses were carried out using ANOSIM analysis with R Vegan package. Correlation (r) and significance (*P*) values are shown for OTU-based and phylogenetic-based distances. Significance values for factors significant at the *P* < 0.05 level are in Bold.

^*^Fire means the effect of fire whether it was burned or not, while *freq fire* separates the treatment of two years burning from other two treatments.

**Table 3:** Mantel correlations between bacterial community structure and soil chemical properties.

variables	Bray-Curtis	
	OTU	dominated OTU*
TC	0.1349(0.105)	0.08597(0.182)
TN	0.1902(0.051)	0.1561(0.067)
Pi	−0.0065(0.456)	−0.04065(0.653)
CN	0.1465(0.084)	0.1221(0.117)
Moisture	0.1295(0.086)	0.07442(0.204)
DOC	**0.3069(0.0006)**	**0.252(0.003)**
DON	**0.2476(0.0019)**	**0.2243(0.009)**
pH	**0.321(0.0002)**	**0.3408(0.001)**
EC	**0.1913(0.0457)**	**0.2493(0.009)**
NO_3_^−^	0.0326(0.3675)	0.08704(0.189)
NH_4_^+^	**0.2105(0.0254)**	**0.1933(0.033)**
MBC	0.1175(0.1413)	0.09893(0.174)
MBN	−0.0196(0.5588)	−0.04852(0.701)

Bacterial community structure was generated from Bray-Curtis (OTU-based) dissimilarity matrices using R with Vegan package. The significances (the number in brackets) are tested on 999 permutations. In order to minimize the rare species for OTUs, we also generated a Bray-Curtis dissimilarity matrix with dominated OTU with the total relative abundance over 0.1% (the first 66 OTUs). Euclidean distances matrix were used for soil chemical properties. Bold values indicate a significant difference at *P* < 0.05. For abbreviations for soil chemical properties refer to Table 1.
