# Supplementary material for: Long term repeated fire disturbance alters soil bacterial diversity but not the abundance in an Australian wet sclerophyll forest
Source: Sci Rep. 2016 Jan 20;6:19639. doi: 10.1038/srep19639 (PMC4726133; doi:10.1038/srep19639)
Supplement: Supplementary Information [file srep19639-s1.doc]

# Long term repeated fire disturbance alters soil bacterial diversity but not the abundance in an Australian wet sclerophyll forest

Ju-pei Shen^1, 2^, CR Chen^1*^, Tom Lewis ^3^

*^1^Environmental Futures Research Institute and Griffith School of Environment, Griffith University, Nathan QLD 4111, Australia*

*^2^Research Centre for Eco-Environmental Sciences, Chinese Academy of Sciences*

*Beijing, China*

*^3^Horticulture and Forestry Science, Department of Agriculture, Fisheries and Forestry,* *University of the Sunshine Coast, Sippy Downs Drive, Sippy Downs, QLD 4556, Australia*

*Corresponding author: [c.chen@griffith.edu.au](mailto:c.chen@griffith.edu.au)

**Supplementary Materials**

**Table S1** Total raw and final sequence numbers for each treatment at two depths

| Depth | Treatment | Sample | Total raw sequences | Final sequences | OTUs  3%cut-off |
| --- | --- | --- | --- | --- | --- |
| Top soil  0-10 cm | 2-year  Burning  (B2) | PC1 **^a^** | 23557 | 16265 | 687 |
|  |  | PC3 | 23747 | 15550 | 812 |
|  |  | PC13 | 21065 | 14341 | 622 |
|  |  | PC15 | 27953 | 18210 | 699 |
|  | 4-year  Burning  (B4) | PC9 | 5585 | 2753 | 630 |
|  |  | PC11 | 7398 | 4568 | 590 |
|  |  | PC17 | 22317 | 15865 | 572 |
|  |  | PC19 | 17738 | 12148 | 555 |
|  | No burning  (B0) | PC5 | 13953 | 9624 | 586 |
|  |  | PC7 | 16393 | 11428 | 620 |
|  |  | PC21 | 13713 | 9305 | 589 |
|  |  | PC23 | 13171 | 9778 | 493 |
| Sub-surface soil  10-20 cm | 2-year  Burning  (sB2) | PC2 | 12061 | 8232 | 527 |
|  |  | PC4 | 21335 | 14134 | 678 |
|  |  | PC14 | 25773 | 17759 | 538 |
|  |  | PC16 | 28498 | 16988 | 818 |
|  | 4-year  Burning  (sB4) | PC10 | 840 | 411 |  |
|  |  | PC12 | 18798 | 12199 | 524 |
|  |  | PC18 | 21488 | 14895 | 508 |
|  |  | PC20 | 15199 | 10825 | 440 |
|  | No burning  (sB0) | PC6 | 20889 | 14389 | 553 |
|  |  | PC8 | 21569 | 14282 | 582 |
|  |  | PC22 | 20366 | 13887 | 537 |
|  |  | PC24 | 25272 | 17760 | 464 |

**^a^** Sample name for each treatment, PC means Peachester site, and the number after PC means the plot number.

**Table S2** Spearman`s correlations between soil chemical properties and relative abundance of different phylum

|  | **TC** | **TN** | **Pi** | **C:N** | **Moisture** | **DOC** | **DON** | **pH** | **EC** | **NO_3_^-^** | **NH_4_^+^** | **MBC** | **MBN** |
| --- | --- | --- | --- | --- | --- | --- | --- | --- | --- | --- | --- | --- | --- |
| *Proteobacteria* | 0.083 | -0.012 | 0.074 | 0.144 | -0.126 | 0.348 | 0.332 | 0.179 | 0.075 | -0.163 | 0.280 | 0.330 | 0.152 |
| *Acidobacteria* | 0.276 | 0.310 | -0.142 | -0.113 | 0.210 | 0.144 | 0.148 | -0.308 | 0.080 | 0.166 | -0.043 | 0.023 | -0.202 |
| *Verrucomicrobia* | -0.301 | -0.209 | 0.022 | -0.119 | -0.069 | **-0.500^a^** | **-0.520** | 0.018 | -0.115 | 0.182 | -0.328 | **-0.432** | -0.092 |
| *Alphaproteobacteria* | -0.071 | -0.162 | -0.015 | 0.262 | -0.281 | 0.222 | 0.235 | 0.226 | -0.073 | -0.260 | 0.133 | 0.194 | -0.050 |
| *Betaproteobacteria* | 0.205 | 0.139 | -0.038 | 0.050 | 0.056 | 0.339 | 0.347 | 0.138 | 0.120 | -0.115 | 0.308 | 0.333 | 0.292 |
| *Deltaproteobacteria* | 0.285 | 0.240 | 0.185 | -0.081 | 0.110 | 0.413 | 0.297 | -0.042 | 0.288 | 0.126 | 0.427 | 0.344 | 0.270 |
| *Gammaproteobacteria* | 0.406 | 0.388 | 0.268 | -0.312 | 0.271 | **0.725** | **0.610** | -0.325 | **0.503** | 0.241 | **0.671** | **0.616** | 0.419 |
| *Bacteroidetes* | **0.489** | **0.501** | 0.078 | -0.345 | 0.341 | **0.752** | **0.665** | -0.292 | **0.519** | 0.209 | **0.756** | **0.645** | **0.645** |
| *Planctomycetes* | -0.113 | -0.170 | -0.095 | 0.216 | -0.072 | -0.216 | -0.094 | -0.066 | -0.172 | -0.099 | -0.350 | -0.148 | -0.214 |
| *Actinobacteria* | 0.146 | 0.057 | 0.153 | 0.145 | -0.087 | 0.404 | 0.375 | 0.248 | -0.062 | -0.315 | 0.324 | 0.311 | 0.151 |
| *Firmicutes* | -0.096 | -0.108 | 0.318 | -0.114 | -0.042 | -0.018 | -0.091 | -0.202 | 0.329 | 0.300 | 0.175 | 0.042 | 0.130 |
| *Chloroflexi* | **-0.486** | **-0.543** | -0.297 | **0.491** | -0.350 | **-0.806** | **-0.702** | **0.575** | **-0.582** | **-0.446** | **-0.766** | **-0.714** | **-0.568** |

a:Bold values indicate a significant difference at *P* < 0.05

Abbreviation for soil chemical properties refer to Table 1.

**Fig. S1** Rarefaction curves showing the microbial biodiversity of the three treatments. For each sample, the minimum number of sequence (2753) was randomly selected to rarefy the number of the sequences. The average values of the three treatments at the depth of 0-10 cm (A) and 10-20 cm (B) were shown with lines. Solid, dash and dash dot lines represent the treatments of burning every two years (B2 and sB2), no burning (B0 and sB0) and burning every four years (B4 and sB4), respectively. Error bars are used to indicate standard deviation for each treatment in respective colour.

**Fig. S2** Soil bacterial 16S rRNA gene copy numbers under different burning frequency at two depths in the year of 2005 and 2010. B0, B2 and B4 represent the 0-10 cm samples from the treatments of no burning, burning every two years and burning every four years, respectively, while sB0s, sB2 and sB4 represent the 10-20 cm samples.

**Fig. S3** Relative abundance of different bacterial taxonomic groups in soils. Top panel: number of sequences in all soil samples assigned to different bacterial taxonomic groups; bottom panel: number of OTUs assigned to different bacterial taxonomic groups
